# Challenges to the control of *Mycobacterium bovis* in livestock and wildlife populations in the South African context

**DOI:** 10.1186/s13620-023-00246-9

**Published:** 2023-07-25

**Authors:** Sewellyn Davey

**Affiliations:** Private Veterinary Consultant, Malmesbury, South Africa

**Keywords:** *Mycobacterium bovis*, Cattle, African buffalo (*Syncerus caffer*), Wildlife, Control, Legislation

## Abstract

Bovine tuberculosis (bTB) was first diagnosed in cattle in South Africa in 1880 and proclaimed a controlled disease in 1911. Testing of cattle for bTB is voluntary and only outbreaks of disease are reported to the National Department of Agriculture so the prevalence of the disease in cattle is largely unknown. There is a Bovine Tuberculosis Scheme which is aimed at the control of bTB in cattle but the same measures of test and slaughter, and the quarantining of the property apply to wildlife as well. bTB was first diagnosed in wildlife in a greater kudu in the Eastern Cape in 1928 and has to date been found in 24 mammalian wildlife species. The African buffalo has become a maintenance host of the disease, which is considered endemic in the Kruger National Park, the Hluhluwe-iMfolozi Park and the Madikwe Game Park. Control of bTB at the wildlife-livestock interface is difficult because of spill-over and spill-back between species. Only buffalo are required by law to be tested before translocation, but bTB has been introduced to the Madikwe Game Park probably by the translocation of other infected wildlife species. There is no national control strategy for the control of bTB in wildlife. Indirect tests have been developed to test for bTB in eight species, 6 of which can be considered endangered. More research needs to be done to develop an effective and efficient vaccine to combat the transmission of bTB within and between species. New policies need to be developed that are effective, affordable and encompassing to control the spread of bTB in South Africa.

## Background

South Africa is a land of beauty and splendour, it is also a land of diversity and contrast. South Africa ranks as the third most biodiverse country in the world with 95,000 known species, 299 of which are mammalian [1]. There are a diverse range of biomes [1]. It has a population of about 60.14 million people [2], 9 provinces and 11 official languages. The Animal Diseases Act, 1984 (Act 35 of 1984) is a national Act but the implementation thereof is a shared responsibility in terms of the Constitution of South Africa between national and provincial governments. Implementation is thus devolved to the nine provinces and is therefore subject to local priorities and resources. 16,3 million people live below the international absolute poverty threshold of $1.90/day [3]. South Africa is urbanising rapidly: 63% of South Africans are already living in urban areas and will rise to 71% by 2030. By 2050, eight in 10 people will be living in urban areas and this will increase demand on basic infrastructure requirements [4].

According to the World Health Organisation (WH0) an estimated 360,000 cases of active human tuberculosis (TB) occurred in South Africa in 2019. The incidence has been dropping since 2009 but TB still remains the leading cause of death in South Africa [5, 6]. In addition, South Africa remains the epicentre of the global HIV pandemic. An estimated 7.2 million South Africans are living with HIV, and in 2019 the HIV co-infection rate among notified TB cases in South Africa was 59% [7].

Climate change is adversely affecting South Africa – dry seasons are becoming longer, and the rainy seasons are starting later and becoming more variable. Torrential downpours as opposed to soft soaking rains cause flash flooding [1, 8]. Much of South Africa is arid or semi-arid and as a result many commercial farmers have switched from livestock farming to wildlife ranching on unproductive and marginal land. There are approximately 9,000 private game ranches owned by 2,000 landowners in South Africa covering an estimated 0,2 million square kilometres. Private game ranches across the country carry roughly three times more wildlife than national and provincial parks [9]. South Africa is home to the “Big Five” i.e., the elephant, rhinoceros, buffalo, lion and leopard which attracts many tourists to the country. Wildlife or eco-tourism is very important to the South African economy as international tourists contribute to the Gross Domestic Product (GDP), job creation, rural development and the socio-economic upliftment of communities surrounding the parks [10].

Cattle farming practices are many and varied in South Africa ranging from commercial farmers, many employing first world standards; to smaller commercial units, emerging farmers, communal (including traditional) farmers and subsistence farmers. Commercial cattle make up around 58% of the national herd, emerging, communal and subsistence cattle 42% of the national herd of 13,601 million cattle. Dairy cattle account for approximately 10% of cattle in the commercial cattle population [11, 12]. There are no subsidies for farmers in South Africa. Over the years profit margins have seen smaller dairies disappear, and the rise of larger dairies where the economy of scale helps keep dairy farms viable. Many of the traditional farming enterprises lie between or alongside game parks, reserves or ranches where wildlife may be infected with bovine tuberculosis (bTB). Spill-over from cattle to wildlife, or spill-back from wildlife to livestock may occur at the wildlife-livestock interface [13–17]. Once endemic in a wildlife population or in a multi-host setting at the livestock-wildlife interface, bTB is very difficult to eradicate.

The Animal Diseases Act, 1984 (Act 35 of 1984) makes allowances for the control and eradication of bTB through its regulations and “Bovine Tuberculosis Scheme R1953”. Testing is voluntary and reporting is poor. Little attention is paid to livestock other than cattle. There is no political will, or funding to support the scheme. There are no subsidies granted for TB testing. The scheme does not differentiate between the control of bTB in cattle or wildlife, or between commercial and communal farmers. There is a socio-economic difference in the value of cattle belonging to commercial and communal farmers which is not considered in the current Bovine Tuberculosis Scheme [13, 15].

The purpose of this rather bleak introduction is to bring into perspective the challenges that can face veterinarians in their quest to either perform a TB test, their interpretation of the TB test once performed; and the control and eradication of the disease.

### Current situation regarding bovine tuberculosis in SA

**Fig. 1:**
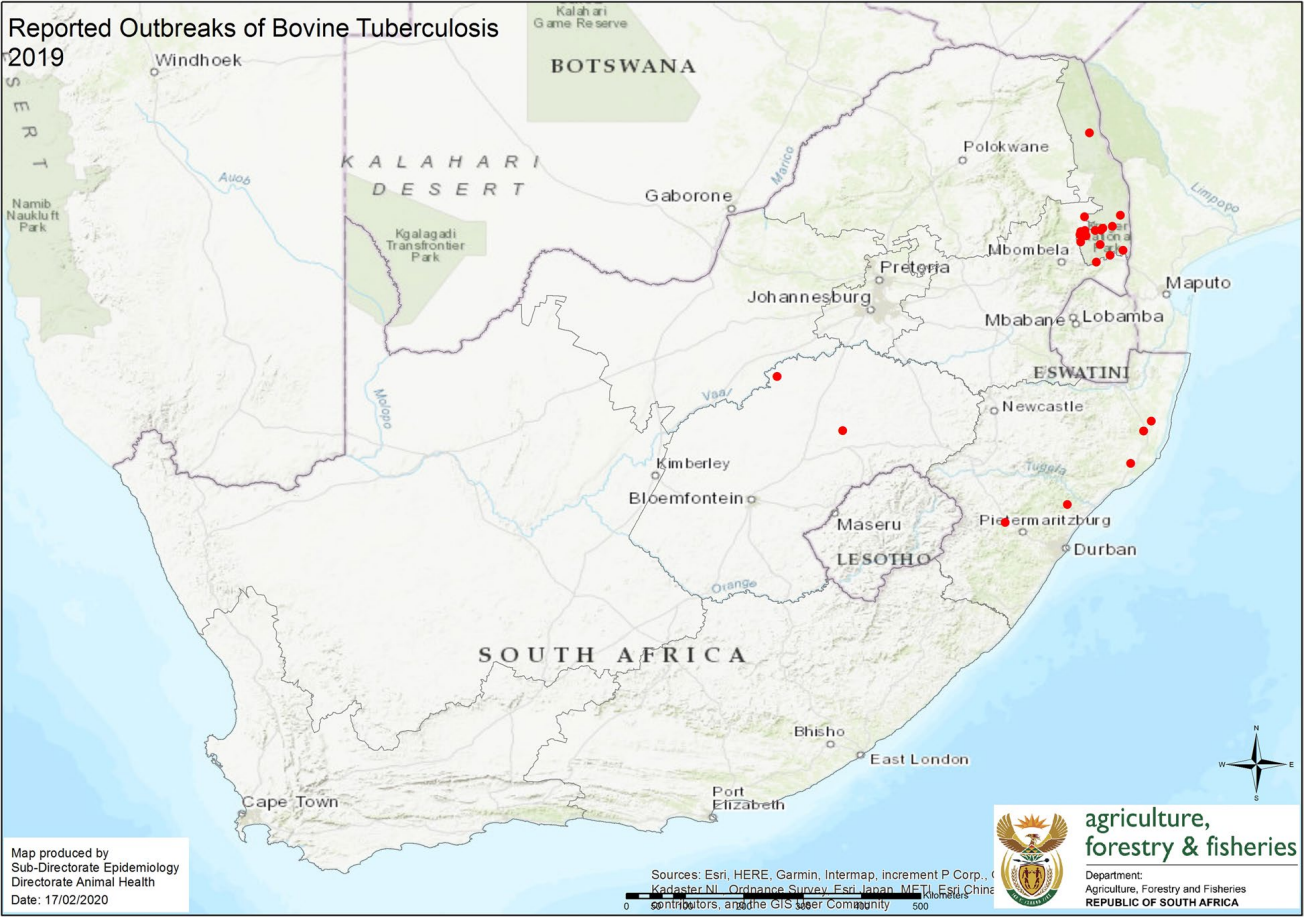
Distribution of reported bTB outbreaks in South Africa during 2019. (Department of Agriculture Forestry and Fisheries) (Dr. Pienaar, Pers. Comm.)

**Fig. 2:**
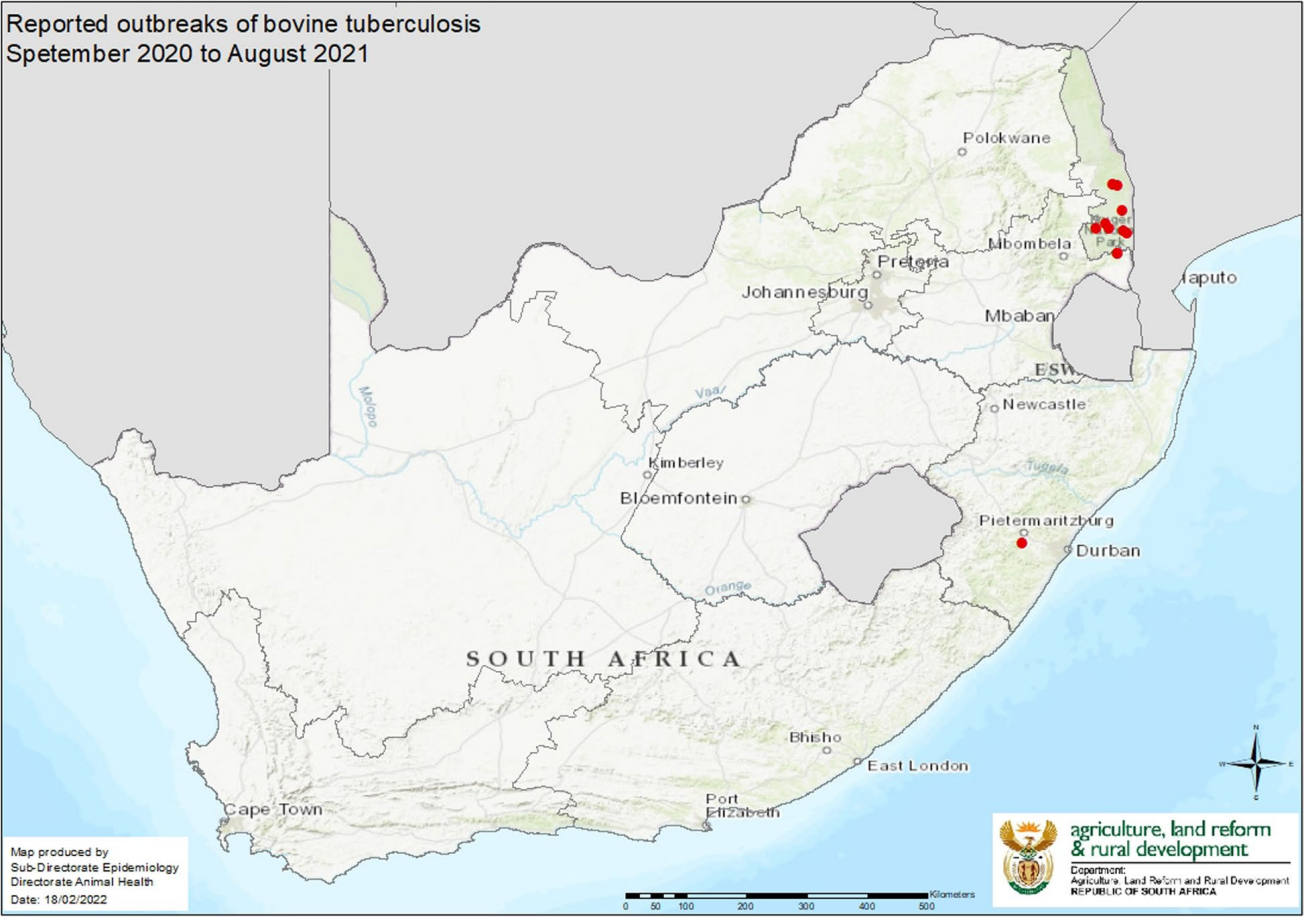
Distribution of reported bTB outbreaks from September 2020 to August 2021. (Department of Agriculture Forestry and Fisheries) (Dr. Pienaar, Pers. Comm.)

Positive skin reactions to the SICTT, or macroscopic lesions indicative of M. bovis infection in culled animals are reported to the epidemiology section of Veterinary Services in Pretoria. The division in turn verifies the data and from this data, Table 1; Fig. 1 for the period January to December 2019; and Table 2; Fig. 2 for the period September 2020 to August 2021 were created (Dr. Pienaar, Pers. Comm.).

**Table 1:** Number of reported bTB outbreaks and cases from January to December 2019. (Department of Agriculture Forestry and Fisheries) (Dr. Pienaar, Pers. Comm.)

Species	No. of outbreaks	No. of cases
Cattle	8	22
Buffalo	10	30
Leopard	5	5
Lion	3	3
Warthog	2	4
Total	28	64

**Table 2:** Number of reported bTB outbreaks and cases from September 2020 to August 2021. (Department of Agriculture Forestry and Fisheries) (Dr. Pienaar, Pers. Comm.)

Species	Number of outbreaks	Number of cases
Cattle	2	6
Buffalo	1	2
Lion	5	5
Total	8	13

During January – December 2019, 28 outbreaks of bTB comprising 64 cases were reported to national government.

Case - means an individual animal infected by a pathogenic agent, with or without clinical signs. Outbreak - means the occurrence of one or more cases in an epidemiological unit. Where an epidemiological unit means a group of animals with a defined epidemiological relationship that share approximately the same likelihood of exposure to a pathogenic agent [18].

Whilst during the Covid-19 pandemic a very different picture emerged.

The prevalence of bTB in both cattle and wildlife is unknown.

### Legislation

There are in essence 3 Acts that provide the legal mandate for control actions related to bTB namely the Animal Diseases Act, The Meat Safety Act and the Animal Identification Act.

Bovine tuberculosis was first noted in cattle in South Africa in 1880 and has been a notifiable animal disease in South Africa since 1911. The “Bovine TB eradication scheme” was introduced in the Republic on 14 May 1969. At that time there was political will as well as sufficient funding of the scheme, and sufficient manpower to implement the scheme. There was a high level of compliance on the part of commercial farmers for the scheme. During 1970/71, 232 Stock inspectors were trained, and private veterinarians were contracted by the state to test for bTB. The state paid compensation at slaughter for any cattle diagnosed on the intradermal skin test as being infected with bTB. When the Animal Diseases Act, 1984 (Act 35 of 1984) replaced the previous Animal Diseases and Parasite Act, 1956 (Act 13 of 1956), tuberculosis was maintained as a controlled disease. The Animal Disease Regulations (GN R2026 of 1986) that was promulgated in terms of Act 35 of 1984, refers to the control of *Mycobacterium bovis, M. avium* and *M. tuberculosis*. As a result of the Act, a voluntary “Bovine Tuberculosis Scheme, R1953” was promulgated on 30 September 1988 and is presently still applicable. There was a uniform computerised reporting system across the four provinces as well as reliable and standardised census data. The bTB Scheme is a voluntary scheme, but once bTB is diagnosed or suspected in a herd, control becomes legally compulsory and a quarantine on the movement of cattle is imposed. There is a Veterinary Procedural Notice for Buffalo disease risk management (Buffalo VPN) for the testing of buffalo for bTB prior to movement. There is no legislative requirement to test other wildlife for bTB.

After 1994 when South Africa became a democratic republic with nine provincial governments, disease control was devolved to the provinces. The control of bTB became a lesser priority with funding prioritised for other priorities within provinces. In accordance of the Animal Diseases Act, control is based on test and slaughter in domestic species and buffalo. Although provision for compensation is made in the Animal Diseases Act, compensation is generally not paid.

According to the Meat Safety Act, 2000 (Act 40 of 2000), no person may slaughter any animal at any place other than an abattoir except if the meat is for their own consumption or for cultural purposes. It is acknowledged that Iillegal or informal slaughtering does take place of which infected TB carcases will not be notified to local authorities. The owner of the abattoir must procure a registered independent meat inspector to perform a meat inspection service. The meat inspection includes *ante-mortem* inspection, primary and secondary meat inspection. Primary meat inspection means the inspection, by a registered meat inspector, of a carcass and organs directly after flaying and evisceration. Secondary meat inspection means the inspection, by a registered veterinarian, of a carcass and organs detained during primary meat inspection such as cattle with visible tuberculous lesions and especially anergic cattle with advanced lesions. The ideal is that when identified during primary meat inspection performed at abattoirs there will be traceability back to the farm of origin.

In terms of the Animal Identification Act, 2002 (Act 6 of 2002), there is a herd identification system where cattle should be branded or tattooed with a unique identification mark supplied by the Registrar of the Act. This Act is policed through the Stock Theft Act, 1959 (Act 57 of 1959) by members of the various Provincial Stock Theft Units, a specialised unit of the South African Police Services. In theory this unique brand mark should be able to trace an animal found to be infected with tuberculosis back to a herd of origin, but in practice it rarely does as animals may not be marked, they may have changed ownership or marks may be unidentifiable. Very few cases of bTB are diagnosed at an abattoir, and if diagnosed are rarely traced back to the herd of origin. There is legislation available to support a national animal identification and traceability system (LITS) in the country under the Animal Identification Act, 2002, but this has not been implemented yet. This will hopefully address one of the challenges of tracing cattle found to be infected with bTB at an abattoir back to the farm of origin.

### Political constraints to eradication

In 1994 South Africa had its first democratic elections. Nine provinces were created, and veterinary services provincialized with central government having a concurrent responsibility with the provinces for animal disease control but in reality, only overseeing and setting of national policies for disease control. Each province has its own focus for animal health and disease control. The OIE summarised the problems arising from provincialisation in their Performance of Veterinary Services (PVS) Evaluation report of the Veterinary Services in South Africa dated October 2012. They reported “Constitutional change has introduced a break in the chain of command in the VS (Veterinary Services) as it has become the “concurrent” responsibility of both national and provincial political authorities. This break in command is universal except in cases of national emergency”. This break in command has also negatively affected disease reporting. The OIE report goes on further to state that “Data management is generally effective and widely utilised. However, the break in the chain of command limits data collection, analysis and reporting at central level. The data is not being used to develop comparative, efficacy, efficiency and cost benefit analyses for animal health programmes” [19].

Currently the National Department of Agriculture does not know how many non-infected herds are being tested for TB as only infected herds are reported by provinces. Because of this and poor census data, the prevalence of bovine TB cannot be established and therefore strategies to implement surveillance or to control and eradicate bovine TB cannot be developed and implemented. Currently there is no national strategic plan for the control and eradication of bTB in livestock or wildlife, and generally no compensation for the slaughter of infected animals.

The breaks in the chain of command negatively affects authority and the manner in which the Bovine Tuberculosis Scheme is implemented in each province. Currently the compliance of livestock owners with the applicable legislation for bTB testing, and the enforcement thereof by government is variable. All bTB testing apart from infected herds and some surveillance cases is paid for by the cattle owner. There is otherwise no funding by industry or the state. Dairy farmers are required by legislation to test their herds for TB, this mainly as a result of export requirements, so the majority of dairy cattle are tested. Some milk buyers will deduct a small amount per litre of milk from payment if the TB and Brucellosis tests are not up to date. This acts as an incentive for the dairy farmer to test. It is thought that most of the estimated 1,4 million dairy cattle in the country are tested for bTB [7]. Stud beef farmers may also test their herds as part of an individual herd health status programme. Commercial beef and communal farmers generally do not test their herds and therefore farm with cattle of unknown health status. However, in some provinces emerging, communal and subsistence farmer’s cattle may be tested by state veterinary services as a form of surveillance; to prevent zoonotic bTB from occurring; or for research purposes [14–17].

Presently there is no clear national implementation plan, or political will to eradicate bTB from cattle herds or wildlife in South Africa. In recent years attention has been focussed on the control of African Swine Fever (ASF), Highly Pathogenic Avian Influenza (HPAI) and Foot and Mouth Disease (FMD) that are draining resources and place exports in jeopardy. As previously stated, the Bovine Tuberculosis Scheme is a voluntary scheme, and implementation of the current legislation is inconsistent and insufficient. National government is focussing on education, social protection and human health, not Agriculture, resulting in a low prioritisation of funding for veterinary service delivery. The Department of Agriculture, Land Reform and Rural Development (DALLRD) only received R27.5 billion of the total budget of R2,157.3 billion in the 2022/2023 budget allocation or 1.2% of the 2022/23 National budget [20]. Programme 2 of DALRRD, to which Veterinary Services belongs received R2.5 billion (9%) of this 2022/2023 budget [21].

Furthermore, there are many vacancies and unfunded posts in State veterinary services. Newly qualified veterinarians are obliged to conduct compulsory community service for the first year after graduating as veterinarians in the employ of national government, either in national government or provincial posts. This is an attempt to fill these vacant positions, not contributing significantly to TB testing. Private veterinarians and Animal Health Technicians (AHTs) working in the private sector are testing cattle and herds, but they are not contracted by the state to do so which leads to poor reporting and low testing frequency. Authorisation of veterinarians and AHTs is needed to stimulate compliance with reporting.

### Technical constraints

*M. bovis* is part of the *Mycobacterium tuberculosis complex* (MTC) that constitutes a remarkably genetically homogeneous group. bTB probably has the widest host range of the pathogenic mycobacteria. MTB and bTB are characterized by a 99.9% similarity at the nucleotide level and identical 16 S rRNA sequences [22]. As stated in the introduction, South Africa has an extremely high level of human TB, and urbanisation is increasing, often leading to an increase in urban farming. This can increase the risk of transmission of mycobacteria between cattle and humans (zoonotic transfer) and conversely between humans and cattle (reverse zoonosis) [23–25].

Zoonotic tuberculosis can occur due to closer contact with cattle with a higher potential infection rate in untested animals and the absence of pasteurisation of milk and its products, wound mediated transmission during slaughter, or the consumption of infected undercooked meat. The higher level of poverty, malnutrition and HIV-infection rate decreases immunity in humans making them more susceptible to infection [26]. If cattle are shedding bTB and infect humans, the extent of the zoonotic infections within a community will not be known as the first line TB test used in South Africa is the Xpert ULTRA, which detects the IS6110 insertion element found uniquely in the MTC so this test would not differentiate the between *M. bovis* and *M. tuberculosis*. Confirmatory testing on all cultured mycobacteria using an immunochromatographic test (ICT) based on the detection of IgG antibodies is used. If the ICT is negative then molecular tests are performed to identify the species of the organism, which includes *M. bovis, M.caprae and BCGosis*. These cases are extremely rare and given the burden of *M. tuberculosis* in South Africa this differentiation is not considered a priority (Dr. Vally Moosa, Pers. Comm). Muller et al. [23] made no mention of bTB cases of tuberculosis being found in humans in South Africa. If a person is suffering from zoonotic TB, the treatment used to treat MTB will be effective even though *M. bovis* is resistant to one of the antibiotics used [27].

A reverse zoonosis is also possible as management practices bring cattle into close contact with MTB, and it will sensitise cattle to the bovine Purified Protein Derivative (PPD) tuberculin and interfere with skin test results [28]. This sensitisation is a problem on dairy farms where staff may be suffering from MTB and the expectoration of sputum exacerbates the problem. Even though farm staff may be tested and not be infected with MTB, visitors to the staff may be infected, and paths often take visitors past cattle camps to staff houses. The inability of skin tests to differentiate between bTB and MTB is very challenging in the field. MTB was isolated from two epidemiologically unrelated farms in the Eastern Cape Province [24]. *M. tuberculosis* was cultured as a co-infection with *M. bovis* from tonsillar tissue in a buffalo from the Northern Cape Province [28]. *M. tuberculosis* has been cultured from a Chacma baboon living in the wild (personal experience) and in a wild elephant in the Kruger National Park (Dr. de Klerk-Lorist, Pers. Comm).

Infected cattle with advanced pathology due to bTB and which are more likely to be shedding *M. bovis*, may not be responsive on the skin test (anergic) and remain in the herd spreading disease [29–31]. The ELISA test may be helpful in antibody detection in these anergic cattle, but its sensitivity may not be sufficient to inform a correct diagnosis [20]. The ELISA test was validated in South Africa but is not generally used.

South Africa does not manufacture its own bovine or avian tuberculin PPD, so this needs to be imported through an import permit acquired from the registrar of the Fertilizers, Farm Feeds, Seeds and Remedies Act, 1947 (Act 36 of 1947). Often this permit can take months to acquire leading to a shortage of tuberculin in the country, and a halt to testing. Currently bovine tuberculin PPD of two manufacturers can be imported into the country – from Intertest (through MSD), and Prionics (through Onderstepoort Biological Products - OBP). Intertest’s concentration is 50,000 IU/ml [32], and Prionics is 30,000 IU/ml [33], both above the OIE Terrestrial Manual’s recommendation of 2,000 IU/dose [28]. However, the difference in concentration between the two PPD’s is noticeable in the field 72 h after testing using the single intradermal tuberculin test (SITT) [34]. Many more small, hard, circumscribed and loose lesions are felt on palpation when the higher concentration of bovine PPD is used. This is particularly unnerving for inexperienced veterinarians or technicians. During the Covid-19 outbreak in 2020, the OBP did not have stock of tuberculin and could not import Bovine PPD due to the lack of international flights, so could not supply veterinarians with Bovine PPD (Dr. Malahlela, Pers. Comm.). MSD who had Bovine PPD reported a significant drop in Bovine PPD sales (Dr. Austin, Pers. Comm.).

Both PPD tuberculin should be stored at a temperature of 2–8˚ Celsius, but Prionics PPD may be transported at 2–37˚ Celsius for up to 14 days which may lead to misinterpretation by the end user [33]. The storage temperature when testing in the field is often not feasible under South African situations as temperatures often exceed the critical levels. Cooler boxes and ice packs cannot maintain the required temperature levels over the course of a day. Most vehicles are not equipped to run small camping refrigerators. In rural areas, it sometimes requires a long walk to get to cattle herds as no roads exist, or are only accessible by 4 × 4 vehicles.

### Non-technical constraints

In dairy herds the risk of spread of bTB between cattle increases as not only do dairy cattle congregate at the dairy parlour two to three times a day, but they also congregate around feed bunkers and water troughs. This risk can be influenced by management where some dairies are moving to a system of zero grazing, and herd housing with fans and misters to keep cows cool, creating the optimum environment for the *Mycobacterium* to survive. Water is a scarce commodity in many areas of South Africa and is being recycled and used to clean the concrete aprons in front of the parlour. Other methods of cow management may also negatively impact on disease control. The damp moist conditions found in dairies can allow for the growth of bTB and other Non-Tuberculous Mycobacteria (NTM) which can sensitise the animal and interfere with the skin tests [35, 36]. From personal experience the use of molasses wheels to increase energy consumption and prevent ketosis in the dry cow camp, led to a resurgence of bTB in a chronically infected dairy herd.

In urban and rural areas, cattle are often kept in small camps close to dwellings at night due to the risk of theft or predation, this congregation of animals increases the risk of spread of bTB in an infected herd and contact with MTB and NTM [37]. The cattle of traditional farmers may have the added problem of becoming infected by grazing near to or with infected wildlife at the wildlife-livestock-human interface, sharing contaminated grazing or water sources, congregation at dip tanks or through uncontrolled movement of animals [16].

A novel NTM, *Mycobacterium malmesburyense* was cultured from various places in South Africa from water, nasal and pharyngeal swabs, as well as bovine lymph nodes [11]. Skin reactions on the dairy farm in the Malmesbury district where this species was identified are extremely difficult to interpret – the non-specific skin reactions may or may not be due to this species [35, 36].

As the skin test has to be assessed 72 h after the introduction of PPD, sometimes in the case in emerging, communal or subsistence production systems some or all the animals are not presented for palpation and diagnosis. To try and avoid this situation, incentives are often used e.g. treatment with anti-parasitics to rid the cattle of either internal or external parasites during reading.

In large dairies, individual cow numbers are normally not recorded – numbers will only be recorded when done in conjunction with blood sampling for bovine brucellosis. Numbers of cattle tested are therefore given by the farmer from his computer spread sheets and may not be accurate. There are often duplicate numbers when other herds with the same format for numbering cows are bought in. Quite often cattle may inadvertently not be tested, or not be presented after 72 h as they have changed milking groups or calved down. As milk production drops when dairy cattle are taken out of their routine, farmers get annoyed with the loss of income over the testing period.

Routine or parallel Interferon gamma (IFG) blood tests to determine the presence of bTB in a herd are not routinely done or are not practical because of cattle numbers, distance to laboratories and great expense involved. Recent research conducted using a bacteriophage method combined with phage-PCR demonstrated MTC circulating in blood [38]. This method may be of use in South Africa once further research is concluded in the European Union. The OIE will first need to approve the Actiphage test, and this will be followed by the long process of getting it imported and validated for South African conditions.

Cattle belonging to farmers in urban and deep rural areas are not used to being handled which makes the process of testing and reading tedious. Pens and crushes are often inadequate or absent. Mobile pens and crushes have been purchased in some state veterinary areas to overcome these difficulties. Long distances, setting up and handling times have a negative effect on the efficiency of TB testing and the use of non-thermal stable PPDs. These cattle are often not individually identified. Another challenge encountered is that torrential rains can cause rivers and streams to become impassable or wash away infrastructure so reading the test 72 h later, or doing follow up testing becomes difficult. The harsh summer sunlight causes sunburn on unpigmented skin which can interfere with the interpretation of skin tests.

In some communal areas one must arrange for testing through the tribal chief, headman or community leader. Negotiation is often very complex and time consuming. If any infected animals are found, retesting of the communities’ cattle is challenging as socio-economic dynamics cause resistance to further testing. Cattle may also have been moved to better pastures for grazing during times of drought [15]. Cultural complexities as well as dialect differences further confound communication with rural communities. Control strategies and law enforcement according to the Bovine Tuberculosis Scheme becomes difficult under these situations.

### Wildlife

Bovine TB was reported in South African wildlife as early as 1928 when it was diagnosed in a greater kudu (*Tragelaphus strepsiceros*) and common duiker (*Syvicapra grimmia*) in the Eastern Cape. The first case diagnosed in the Kruger National Park (KNP) was in an impala (*Aepyceros melampus*) in 1967. In 1970 bTB was diagnosed in a black rhinoceros (*Diceros dicornis*) in the Hluhluwe-iMfolozi Game Park (HiP) in Kwa Zulu Natal. During 1990 bTB was diagnosed in the African buffalo (*Syncerus caffer*) in the south of the KNP and has since spread to the north of the park and into neighbouring Zimbabwe. bTB has also been diagnosed on private game farms and ranches. The presence of well-established national and provincial game parks as well as the increasing development of wildlife ranches in South Africa, is increasing the wildlife-livestock interface and risk of either wildlife contracting disease from livestock (spill-over) or livestock contracting disease from wildlife (spill-back) [16, 17, 38]. The translocation of various game species between game parks and ranches increases the risk of spreading disease as only buffalo are required by law to be tested before translocation. To date *M.bovis* has been diagnosed in 24 of the 246 terrestrial mammalian wildlife species in South Africa [39].

Wildlife host species can be described as maintenance hosts where infection can persist without introduction from another source, or spill-over hosts when species become infected incidentally [16, 17]. As bTB is considered an alien infection of African wildlife, hosts are naïve, and infections are often devastating to the animal concerned. bTB has a wide host range and in the absence of a vaccine once established in a wildlife population it is almost impossible to eradicate the disease. There is no policy document aimed specifically at the control of bTB in wildlife as the Bovine Tuberculosis Scheme is aimed specifically at domestic species but is applicable to wildlife species. This approach is not scientifically defendable. The HiP has a management policy in place to try and keep the prevalence of bTB in its buffalo herds below 10%. This is based on mass capture, tuberculin skin testing and removal of positive animals. This programme has been successful [13, 31]. The KNP monitors the situation through surveillance projects to determine the distribution and rate of spread of disease [13]. Culled game pass through the abattoir at Skukuza where primary meat inspection will show carcases infected with bTB. There was also a successful buffalo breeding programme between 1998 and 2011 to preserve the genetics of the KNP buffalo (Dr. de Klerk-Lorist, Pers. Comm.). The Madikwe Game Reserve planned a surveillance strategy as well as a buffalo salvage plan whereby they aimed to establish a disease-free buffalo breeding herd (Dr. de Klerk-Lorist, Pers. Comm.).

African buffalo movement is strictly controlled by the Buffalo VPN (Veterinary Procedural Notice) with Single Intradermal Comparative Tuberculin Test (SICTT) testing required before movement between farms or game parks with negative results. Each farm or park owner that wants to keep buffalo must apply for registration with national government prior to receiving buffalo, in terms of Regulation 20 A (2) of the Animal Diseases Act, 1984 (Act No. 35 of 1984) as published in Government Notice No. R. 2358 of 10 December 1993, and if approved the farms/parks will receive an individual BU (buffalo) registration number. All buffalo must be microchipped before testing to ensure individual identification and traceability. Ear tags may be used in conjunction with microchips for easier identification. Pre movement testing of buffalo requires testing for *M. bovis, Brucella abortus*, Foot and Mouth disease (FMD) as well as Corridor disease caused by the protozoan *Theileria parva lawrencei*. All four diseases are transmittable from buffalo to cattle, and freedom from these diseases is to prevent spread of disease, and protect other buffalo farms as well as cattle at the wildlife-livestock interface. Buffalo needs to be chemically immobilised for testing and confined in a boma. Confinement in bomas when testing may bring buffalo into contact with NTM which may influence the outcomes of the SICTT [31, 37]. If a buffalo is diagnosed as being positive for bTB, compliance by owners regarding its destruction is often met with stiff resistance due to the value of the animal, and the indefinite quarantining of the farm/parks [15]. Often a parallel test using the interferon-gamma test will be requested by the owner. This comes at a great expense as the buffalo need to be chemically immobilized and the blood transported over great distances to a participating laboratory. Testing of other wildlife that may harbour bTB is not required and translocation between game farms or parks has introduced bTB to previously non infected farms/parks [39]. Since March 2017 this quarantine includes all species susceptible to bTB and these species may not be allowed off the property.

The African buffalo is the most widely known maintenance host of bTB in South Africa [17]. They are herd animals and transmission of bTB is thought to be primarily by aerosol droplet spread and further aided by the animal’s social structure and nature. Herds tend to be large and can consist of hundreds of buffalo. Young adult males migrate between herds, and herds combine on a seasonal basis allowing for the transfer of infection between herds. Periods of drought may change herd dynamics and increase the spread of bTB between individuals due to increased intra and inter species contacts, while physiological and social stress may increase the rate of pathogenesis of disease within an individual [13].

The greater kudu has a social structure, but herds are very small ranging from 3 to 15 individuals with males often being solitary individuals, joining the herd during the mating season. Kudu are browsers so they are primarily infected via the oral route with initial pathology in the lymph nodes of the head where abscessation occurs. Despite its smaller herd size, the greater kudu has the recently been characterised as a maintenance host [13, 15] (Prof. Michel, Pers. Comm.).

bTB has been confirmed in other South African ruminants including impala, springbok (*Antidorcas marsupialis*) bushbuck (*Tragelaphus scriptus*), nyala (*Tragelaphus angasii*), blue wildebeest (*Chonnochaetes taurinus*), eland (*Taurotragus oryx*), and giraffe (*Giraffa camelopardelis*).

Warthogs (*Phcochoerus africanus*) and bushpigs (*Potamochoerus porcus*) are omnivorous and have been found to be infected with bTB. Warthogs live in burrows and are social animals. They become infected via the aerosol droplet route or *per os* when scavenging [15]. They have the potential to become maintenance hosts in high population densities [13, 15]. Their distribution is increasing in South Africa and as they can burrow out of fenced properties their potential for spreading bTB at the wildlife-livestock interface should not be underestimated.

Predators such as lion (*Panthera leo*), leopards (*Panthera pardus*), cheetah (*Acinonyx jubatus*) and wild dog (*Lycaon pictus*) have been found to be infected with bTB [13, 15]. Lions are social animals and hunt in packs. They kill their prey by smothering them so aerosol droplet infection is one of three ways in which they can become infected. The second is through the alimentary canal when eating an infected carcass (*per os*), and the third is percutaneous when fighting over an infected kill. Lions have the potential to become a maintenance host [13]. The rest of the predators can be considered as spill-over hosts and not important in the maintenance of the disease within their population. The potential increase of the disease in the critically endangered wild dog and cheetah is of great concern.

Other wildlife that has been found infected with bTB are elephant (*Loxodonta africana*), white rhinoceros (*Ceratotherium simum*), large spotted genet (*Genetta tigrina*), banded mongoose (*Mungos mungo*), Chacma baboon (*Papio ursinus*), honey badgers (*Mellivora capensis*) and hippopotamus (*Hippopotamus amphibius*), and are all considered spill-over hosts [19]. Again, bTB in the endangered rhinoceros is of grave concern.

Indirect diagnostic tests to detect cell mediated immunity to *M. bovis* in eight species of wildlife have been developed [40]. These tests will prove vital in the early diagnosis of bTB in wildlife populations and may pave the way for the development of diagnostic tests in other species. A trial using the ELISA test was performed on buffalo in the HiP, but results were disappointing as the ELISA did not identify a significant number of anergic animals [31].

Vaccination can be a method for controlling the spread of bTB by increasing an animal’s immunity to disease. Vaccination does not necessarily have to prevent infection but should reduce transmission in the host species and reduce spread to other species. There are many challenges to the successful development of an effective vaccine. A trial done on yearling buffalo in the KNP using a BCG vaccine found that there was no significant reduction in the number of lesions or severity of disease [41]. An efficient and cheap method of vaccination would also need to be developed. A DIVA (Distinguishing Infected from Vaccinated Animals) would also have to be developed for testing vaccinated animals if a live vaccine is used. If an inactivated vaccine is used the Actiphage test could be of use if proven to be of diagnostic value [38].

bTB is of great significance in wildlife as it is a threat to South Africa’s rich biodiversity with the possibility of localised extinction of some species where mortality is high, and population is low. This will have a serious impact on ecosystems as each species fulfils a function in an ecosystem [13, 15]. There is the possibility of spread of disease to other species as well as spill-back to livestock and humans. Prevention of the spread of bTB to new areas by pre-movement testing of known maintenance or potential maintenance hosts would be ideal. The Biodiversity Finance Initiative (BIOFIN) has proposed a “Development and implementation of a voluntary market-based certification scheme in the wildlife sector” but unfortunately animal disease is not mentioned in the proposal [42].

Eradication by test and cull in low prevalence herds/groups of animals would slow down the spread of the disease and may not elicit such an emotional outcry as blanket culling. However, this remains a challenge in large buffalo herds in the KNP [13]. Both testing prior to movement, and test and cull methods require that there are validated tests with acceptable sensitivity and specificity levels in order to be successful. Costs are the most important inhibiting factor for testing wildlife. These costs include the method of capture, chemical immobilisation, manpower and tests. The use of vaccination is another possibility to control bTB in wildlife although methods to vaccinate different species successfully will be challenging. Fortunately, there is ongoing research into bTB in wildlife in South Africa and some indirect tests have been validated for eight species [40]. There is no mass migration of wildebeest and zebra as experienced in Kenya, which could have been an important spreader of bTB.

## Conclusion

In conclusion, bTB is a problem both in cattle and wildlife in South Africa with the potential to reinfect both cattle and wildlife at the wildlife-livestock interface. The extent of bTB in humans is unknown as no differentiation between MTB and bTB occurs routinely. bTB can be treated successfully in humans although some drug resistant bTB forms may occur [27]. The prevalence of bTB in the cattle population is not known due to lack of reliable census data, testing, reporting and adequate policy and political will to eradicate the disease. From reporting over the years, it is believed to be of a low prevalence in most areas [25]. Cattle testing will continue to rely on the SITT as gamma interferon serological tests are generally not an option due to cost, paucity of laboratories performing these tests, and time and temperature constraints involved. MTB interferes with the interpretation of skin tests in cattle and can be challenging at the livestock-human interface. An affordable, reliable and rapid test to differentiate between MTB and bTB in cattle to improve diagnosis would be of great benefit in a country where the incidence of human TB is so high, and the human-livestock interface is increasing. bTB in wildlife has unique and complex challenges which requires much more input from researchers, conservationists and policy makers.

## Data Availability

Not applicable.

## Abbreviations

AHT: Animal Health Technician
ASF: African Swine Fever
BIOFIN: The Biodiversity Finance Initiative
bTB: Bovine Tuberculosis
DALLRD: Department of Agriculture, Land Reform and Rural Development
DIVA: Distinguishing Infected from Vaccinated Animals
FMD: Foot and Mouth Disease
GDP: Gross Domestic Product
HiP: Hluhluwe-iMfolozi Game Park
HIV: Human Immunodeficiency Virus
HPAI: Highly Pathogenic Avian Influenza
ICT: Immunochromatographic Test
KNP: Kruger National Park
LITS: Livestock Identification and Traceability System
MTC: Mycobacterium Tuberculosis Complex
MTB: Mycobacterium tuberculosis
MSD: Merck Sharp and Dohme
NTM: Non-Tuberculous Mycobacteria
OBP: Onderstepoort Biological Products
OIE: World Organisation for Animal Health
PPD: Purified Protein Derivative
SICTT: Single Intradermal Comparative Tuberculin Test
TB: Tuberculosis
VPN: Veterinary Procedural Notice
VS: Veterinary Services
WHO: World Health Organisation
